# Circulars, Bulletins and Reports Issued from the Office of the Chief Surgeon of the American Expeditionary Forces in France

**Published:** 1919-01

**Authors:** 


### CIRCULARS, BULLETINS AND REPORTS

Issued from the Office of the Chief Surgeon of the American Expeditionary Forces in France.

*Under this heading will be published extracts from circulars and bulletins issued by the Chief Surgeon of the Medical Department of the American Expeditionary Forces in France. It is believed that these will be of general interest and value to medical officers.*

### EXTRACTS FROM C. S. O. CIRCULARS

#### Diphtheria and Diphtheria Carriers in the Army

I. *Bacillus Diphtheriae.* (a) True diphtheria bacilli when freshly isolated and examined in young cultures (24 hours on Loeffler's Blood Serum) have fairly typical morphology and staining reactions, which usually serve to differentiate them from other organisms.

(b) Their positive identification may be made upon morphology and staining reactions plus cultural characteristics.

(c) B. diphtheriae may be divided into two groups : virulent and avirulent, which are indistinguishable from each other morphologically, tinctorially and culturally, but may be positively differentiated by guinea pig inoculation.

(d) Practically speaking, an avirulent strain of diphtheria bacilli never acquires virulence, and a virulent strain retains its virulence with great tenacity.

II. *Etiology.* Clinical diphtheria is produced only by virulent diphtheria bacilli.

III. *Diphtheria Bacillus Carriers.* (a) Single throat cultures from healthy individuals of various ages reveal B. diphtheriae in 1 per cent. to 30 per cent. The average incidence appears to be 3 to 4 per cent.

(b) Among the bacillus carriers the per cent. of carriers with virulent bacilli varies greatly, but is commonly found to be 10 to 15 per cent. of carriers.

(c) The carrier state may be temporary or chronic. Sometimes diphtheria bacilli disappear from the throat of a carrier within a few days after they find lodgment there; in other cases they persist for weeks, months, or even years.

(d) If daily cultures are taken from the throats of chronic carriers, very interesting and instructive results may be obtained : (1) Positive cultures may be obtained for a number of consecutive days, extending perhaps over weeks. (2) A majority of the cultures may be positive, with occasional negatives interspersed among the positives. (3) A majority of the cultures may be negative with occasional positive cultures. (4) A carrier who has been giving regularly positive cultures for a number of days may show irregular results for a time and then give entirely negative cultures for a number in succession, to be followed still later by regularly positive cultures, and this condition of affairs may repeat itself many times. (5) The growth of diphtheria bacilli is not confined to the surface of the mucous membrane; colonies have been demonstrated in the depths of the tonsillar tissue, and the condition described under (4) above is probably to be explained by the successive coming to the surface of these deep colonies as the superficial layers of the tonsils are gradually exfoliated. (6) Virulent and avirulent bacilli are rarely, if ever, found in the throat of a carrier at the same time.

IV. *Sterilization of Carriers.* To free carriers of virulent diphtheria carriers, a great number of methods have been tried. The only one which has met with any considerable degree of success in chronic carriers has been tonsillectomy. This will not prove universally successful, as in some cases the nidus may be elsewhere than in the tonsils, as, for example, in the accessory sinuses.

V. *The Role of Carriers in the Spread of Diphtheria.* The role of carriers who have not been in close contact with an active clinical case of diphtheria in the spread of diphtheria does not seem to be important. This is obvious when it is recalled that 85 to 90 per cent. of all carriers harbor only non-virulent bacilli, and that infection does not readily occur from the remaining 10 to 15 per cent. who constitute a possible source of infection for susceptible individuals.

VI. *The Detection of Carriers.* A single throat culture from any large number of people would probably reveal less than half the actual number of carriers present. Two cultures, taken with an interval of a week or two between, would probably reveal twice the number of carriers found on a single culturing. If six or seven cultures were taken with an interval of a week or two between cultures, the number of carriers remaining undiscovered would probably be very small. Nasal cultures might show a few additional carriers, but very few.

Isolation of healthy carriers is impracticable because (1) of the labor involved in detecting all the carriers. (2) If all the carriers among any large group of persons were detected their number would be too great. (3) The only method of sterilizing chronic carriers (tonsillectomy) that has met with much success could hardly be recommended as a routine procedure, and without this many of them would remain carriers indefinitely. (4) They do not constitute a menace serious enough to justify any of the above procedures. (5) Finally, if for any reason an attempt is made to detect and isolate carriers, virulence tests should be performed and the carriers of avirulent organisms should be disregarded.

VII. *The Diphtheria Patient.* While the healthy carrier of even virulent diphtheria bacilli does not constitute a serious danger to persons in contact with him, the same cannot be said of the individual suffering from clinical diphtheria. The disease is readily transmissible, both by direct contact and by moist discharges from the nose and mouth. Strict isolation of all cases should be carried out and thorough disinfection of all clothing, bedding and other articles that have been used by the patient subsequent to his infection. It is possible that persons who have recently become carriers by contact with a diphtheria patient may be a greater source of danger in the spread of the disease than the ordinary healthy carrier who has not been recently in contact with the disease; therefore, all those who are in intimate contact with a person at the time of, or just prior to, his development of diphtheria should be isolated until the incubation period of the disease has passed, or until they can be shown to be free from the infection by at least two negative throat cultures. All nurses and orderlies in attendance upon cases of diphtheria should be isolated during the whole of the time that they are in charge of such patients, and for a period thereafter equal to the incubation period of the disease, or until they are shown free from the infection by at least three successive negative throat cultures at intervals of three days.

VIII. *The Incubation Period.* The incubation period of diphtheria is from two to five days, oftenest 2 days, and under experimental conditions has been found to be as short as 24 hours.

IX. *Treatment with Diphtheria Antitoxin.* Diphtheria antitoxin given in adequate doses sufficiently early in the disease will effect a prompt cure in practically 100 per cent. of cases. There should be no mortality where antitoxin is given within 24 hours of the development of symptoms. For adults weighing 90 pounds or over the amount of antitoxin required in the treatment of cases is as follows:

| | |
|---|---|
| Mild Cases. . . . . . . . . . . . | 3,000 to 5,000 units. |
| Moderate. . . . . . . . . . . . . | 5,000 to 10,000 units. |
| Severe* . . . . . . . . . . . . . | 10,000 to 20,000 units. |
| Malignant* . . . . . . . . . . . | 20,000 to 40,000 units. |

* When given intravenously one-half the amounts stated.

Cases of laryngeal diphtheria, moderate cases seen late at the time of the first injection. and cases of diphtheria occurring as a complication of the exanthemata should be classified and treated as " severe " cases.

In all cases a single dose of the proper amount, as indicated in the schedule, is recommended.

It is recommended that the methods of administration be as follows :

Mild Cases — Subcutaneous or intramuscular.

Moderate Cases — Intramuscular or subcutaneous..

Severe Cases — Intramuscular or subcutaneous or intravenous.

Malignant Cases — Intravenous or intramuscular.

Some point on the surface of the body should be chosen for the injection, as where there is an abundance of subcutaneous cellular tissue, the abdomen or infrascapular region. Before the remedy is administered, the skin should be sterilized at the point of injection with tincture of iodine or other disinfectant. The syringe should be thoroughly sterilized. It is better not to employ massage over the point of injection.

### The Early Administration of Antitoxin.

The earlier the remedy is administered the more certain and rapid is the effect. In cases of any severity where diphtheria is suspected, it is far better to administer the remedy at once, making a culture at the same time, than to delay the treatment until a diagnosis has been made by bacteriologic examination. The first injection should be large enough to control the disease. One large dose given early is far more efficacious than the same amount in divided doses. Severe cases and those in which the administration of antitoxin has been delayed, or cases which are progressive because of an insufficient first dose, should receive a large intravenous injection whenever feasible. In this way the full value of antitoxin is obtained at once, whereas the absorption from the subcutaneous injection is so slow that many hours must elapse before any great amount of antitoxin has found its way into general circulation. It must be warmed to the body temperature and given very gradually.

X. *Anaphylaxis.* While it must be admitted that anaphylactic shock may follow the administration of diphtheria antitoxin serum, and that this danger is slightly greater when the serum is given by the intravenous route than when given subcutaneously or intramuscularly, instances of serious consequences from therapeutic use of diphtheria antitoxin are so rare that there is no justification in with-holding antitoxin in clinical diphtheria. Desensitization may with advantage be attempted in cases of known sensitiveness to horse serum.

XI. *Immunity.* A. Natural immunity. Experience has shown that approximately 50 per cent. of mankind are naturally immune against diphtheria, This immunity is due to the presence, naturally, of a small amount of diphtheria antitoxin circulating in the blood. This immunity once established apparently lasts throughout life. — *The Schick Test.* The presence of natural or artificial immunity may be determined by the Schick test. This test consists in the intradermal injection of a small amount of diphtheria toxin; if antitoxin is present (natural immunity) the toxin injected will be neutralized and no reaction will follow. If no antitoxin is present (as in a susceptible individual) the toxin will give rise to an inflammatory reaction at the site of inoculation, a positive reaction. Technic of the Schick test. The test consists in the intracutaneous injection of 1/50 M. L. D. diphtheria toxin in volume of 0.1 c. c. The M. L. D (minimum lethal dose) of toxin is that amount which will kill a 250 gram guinea pig in 4 to 5 days. For the injection a 1 c. c. hypodermic syringe with very small sharp needle is necessary, and the injection may conveniently be made into the skin of fore arm.

B. *Susceptibility.* It seems highly probable that people who give a negative Schick test may be exposed freely to diphtheria without danger of their contracting the disease, while persons giving a positive Schick test so exposed are likely to contract the disease.

C. *Active Immunization.* Susceptible individuals may be actively immunized against diphtheria by the injection of toxin-antitoxin mixtures, and such immunity is probably fairly lasting, in some instances persisting throughout life.

D. *Passive Immunization.* Susceptible individuals may be passively immunized against diphtheria by the injection of antitoxin. Such immunity reaches its maximum degree immediately, if the antitoxin is injected intravenously, and after about 48 hours following subcutaneons injection. Passive immunity following the usual prophylactic dose of 1,000 units of antitoxin gives the individual a temporary immunity against natural infection, but the immunity is transitory, diminishing rapidly and usually lost in ten days or two weeks. Rarely persons may retain some demonstrable degree of immunity as long as three weeks. Subsequent use of antitoxin for passive immunity in the same individual develops even a briefer protection.

E. *Prophylactic Use of Antitoxin.* Experience has abundantly demonstrated the almost absolute power of a prophylactic injection of antitoxin in preventing the development of diphthéria in persons who have been exposed to the disease. It probably has no effect in preventing the lodgment and growth of bacilli in the throats of such persons, and it is conceivable that the bacilli which have lodged in the throats of such persons might persist and give rise to the disease after the transient immunity conferred by the antitoxin has disappeared. That this frequently happens is not borne out by experience. It is evident, however, from what has been said about natural immunity, that in approximately 50 per cent. of persons there is no need of giving prophylactic injections of antitoxin, since this proportion of humans are naturally immune. If prophylactic injections are to be given it is worth while to perform a preliminary Schick test and give antitoxin only to those who are thus shown to be susceptible by a positive reaction.

XII. *Prevention of Spread of Diphtheria.* Undoubtedly the most important measure in preventing the spread of diphtheria is the prompt recognition of cases as soon as they develop, and effective isolation of them. It is undoubtedly true that many cases are not immediately recognized, and that they give rise to a spread of the disease among their associates.

At a time when diphtheria is prevalent, frequent throat inspections should be made of all individuals exposed, or who may been exposed, and any person having a throat that looks at all suspicious should be isolated and regarded as having diphtheria until negative cultures prove that the suspicion is unfounded. This measure alone, if efficiently carried out, will probably serve to prevent any spread of the disease.

XIII. *A Typical Case of Diphtheria.* It should be borne in mind that infrequently cases of diphtheria occur in which the typical appearance of the throat is lacking, and the symptoms may be so mild that they may be overlooked. The pharynx in these cases may present a beefy red appearance with perhaps a few pinhead sized patches, and the symptoms consist in little more than a feeling of malaise on the part of the patient. The thermometer will usually reveal a slight elevation of temperature, and it is these cases that may escape isolation and, by freely mingling with their associates, give rise to a spread of the disease.

XIV. *Wholesale Measures in Dealing with Epidemics Illogical and Valueless.*

There are certain measures that have become so well established in dealing with epidemics of diphtheria that to question them is sure to arouse the antagonism of those whose ideas have become fixed by tradition. These are the wholesale taking of throat cultures and the prophylactic administration of antitoxin. A knowledge of the practical limitations of application of wholesale culturing to organizations or groups among which diphtheria has appeared, and the poverty of actual results in detecting the insignificant incidence of carriers of virulent B. diphtheriae should suffice to forbid the practice. Similarly, the uselessness of administering diphtheria antitoxin to insusceptibles, and the temporary character of the protection given to susceptibles by passive diptheria immunization, will serve to put an end to the routine of diphtheria antitoxin without Schick reaction control for prophylactic purposes in an organization where diphtheria has appeared.

XV. *Selective Immunization.* We may next consider the advisability of determining the susceptible individuals, either in a camp or among those who presumably have been most exposed to the danger of infection, and of giving prophylactic doses of antitoxin to those persons, or of applying other precautionary measures to them. The susceptible individuals may be discovered by means of the Schick test. The results may be known at the end of 48 hours. If a camp of 5,000 men be tested, 25 per cent. or 1,250 may be found susceptible, and these are the only ones who run any risk of developing diphtheria and to whom the prophylactic injection of antitoxin could be of any use.

If the Schick test is applied to a small group (those who have been more intimately exposed to the disease) one will have to deal with a proportionately smaller number of individuals.

XVI. *Principles for Management of Diphtheria Outbreak.* In all preventive measures the two main objects to be accomplished should be kept clearly in mind : (1) the protection of the individual; (2) the protection of the community. We should also keep clearly in mind what we consider constitutes the danger to the individual, and what to the community.

1. The danger to the individual is that he may develop diphtheria.

2. The danger to the community, as usually considered, is that diphtheria may be spread by : (a) diphtheria bacillus carriers; (b) the failure properly to isolate recognized cases of diphtheria; (c) contact with persons who are in the incubation period of the disease; (d) unrecognized cases of diphtheria with which healthy persons are allowed to come in free contact.

1. *The Danger to the Individual that He May Develop Diphtheria :* Among adults there is a 75 per cent. factor of safety to start with, represented by natural immunity. This is further increased by the chance that of the 25 per cent. of susceptible adults exposed to diphtheria not all of them will have diphtheria bacilli implanted in throats — a chance however that for the sake of safety we will not consider. Of any group of individuals exposed to diphtheria, the susceptible ones may be determined by the Schick reaction. It is obviously unnecessary to give a prophylactic dose of antitoxin to any but the susceptible persons. The time necessary to determine the result of the Schick reaction is 48 hours, and during this period all the contacts should be kept in insolation. The incubation period of the disease is given at " from two to five days, most often two ", so that by the time the result of the Schick test is known most of those who are going to develop the disease will already have manifested signs or symptoms. The Schick test has therefore been unnecessary. Antitoxin given in the first 24 hours of the disease is curative in practically 100 per cent. of cases. Therefore if isolation and observation of the contacts only is employed, without the prophylactic use of antitoxin or the Schick test, the occasional individual who develops the disease under the conditions has lost little, if anything, and the large majority of contacts have experienced no inconvenience other than a very short isolation.

2. *Danger to Community.*

(a) From carriers : There is no danger from the carrier of non-virulent bacilli, and the danger from the ordinary healthy carriers of virulent bacilli is so slight that it does not seem practical to take any measures against it.

(b) The necessity of carefully isolating all recognized cases of diphtheria is so universally acknowledged and practically carried out that no further discussion of this point seems necessary.

(c) That persons in the incubation period of the disease constitute a distinct danger is certain, and the prompt isolation of persons who are in contact with diphtheria cases is an important measure. Fortunately the short incubation period of the disease makes necessary only a very brief isolation. If these contacts are isolated and a daily observation made of their throats and symptoms, no other measures are necessary unless suspicious symptoms arise. In such cases cultures should be made and antitoxin given according to the developments.

(d) Unrecognized cases of diphtheria : It is probable that these cases are the most potent agents in giving rise to the spread of the disease. At a time when diphtheria is prevalent the most important measure, other than the isolation and treatment of the recognized cases of diphtheria, is the search for the mild cases which might otherwise escape detection. Daily inspection of throats with an inquiry as to symptoms will serve to discover all suspicious cases. If these are isolated as they are discovered, a culture taken, and in sufficiently suggestive cases antitoxin given, no serious spread of the disease need be feared. The taking of cultures may be limited in these cases, and to the routine procedure covered by army orders for the discharge of patients convalescent from diphtheria and to those who have been in attendance on diphtheria.

The Schick reaction may be of value in eliminating seventy-five per cent. of the individuals constituting any group as naturally immune, and therefore unnecessary to be kept under observation as possible subjects of diphtheria. It may further be of use in selecting naturally immune persons to serve as attendants on diphtheria patients, and finally if active immunization against diphtheria should be undertaken, it will discover those persons who stand in need of immunization.

---

### EXTRACTS FROM C. S. O. BULLETINS

### Artificial and Acquired Immunity Against Pneumomia.

*(An important opinion from Col. F. F. Russell, M. C.)*

" It is more and more evident to us that acute lobar pneumonia is, under proper circumstances, just as much an epidemic disease as measles. We have had well studied small epidemics occurring among limited groups at certain cantonments in the United States, and hope to have more such studies soon. The reason we get sporadic cases only in the cities has not yet been satisfactorily explained. Our hypothesis, however, is something like this: that the disease in the cities is limited by lack of non-immune susceptible material, and that most city people are protected against lobar pneumonia by having an acquired immunity against most types of the organism, seems most likely. The country boy, no matter how vigorous and healthy, succumbs to infection with the pneumococcus, not because of diminished resistance but because of the lack of active acquired immunity. This, of course, remains to be proven, but it seems to be more and more true that city dwellers are protected by an acquired immunity and not by anything else, the immunity having been acquired in most instances through a temporary carrier state and not through an attack of the disease.

We are sufficiently convinced of the efficacy of the present vaccine to feel that it is worth while to vaccinate all volunteers throughout the United States during the coming winter. In times of peace, we should proceed more cautiously, but in time of war the justification for prompt action is evident.

The grounds for believing that the pneumonias are followed by immunity to the sort of pneumonia from which the patient has suffered are good, and I believe in the end we shall come to recognize that the pneumonias are epidemic diseases, Types I, II, and III of which leave behind a protective immunity. "

Special attention is called to the availability of pneumococcus lipo-vaccine, as detailed in Circular No. 59 from the Chief Surgeon's Office, Dec. 9, 1918.

### Coughing Is Rarely Camouflage.

*Don't Forgel.*

1. To study your chest cases carefully.
2. To employ postural drainage. (See Weekly Bulletin No. 28, and this issue.)
3. That all chest cases must be properly classified on this side of the Atlantic.
4. On arrival in the United States only tuberculosis cases go direct to tuberculosis hospitals.
5. The tuberculosis observation centers in the A. E. F. and the tuberculosis hospitals in the United States should not be flooded with convalescents from grippe and broncho and lobar pneumonia.
6. About 80 per cent. of the cases diagnosed tuberculosis are not tuberculosis.
7. Do your part and do not pass the TB buck. If in doubt obtain a consultant's advice and reach a decision.
8. Post-mortem evidence corroborates the clinicians who find there is exceptionally little incidence of pulmonary tuberculosis in the A. E. F.
9. Repeated negative sputa reports (15 examinations) should make you very suspicious that you are not dealing with tuberculosis.

### Toilet of the Lungs.

The enclosed method of securing " Toilet of the Lungs for Pulmonary Cases, " has been used for several weeks at Convalescent Camp, Allerey, with extreme satisfaction and is readily carried out with large bodies of men as part of the physical training.

The results are eminently satisfactory and in most cases curative.

These exercises are given to convalescents who have been treated in the hospitals for effects of enemy gas, continuation of bronchitis after influenza, and allied conditions.

At the completion of the drills, the Companies being in column of squads, the command is:

" Squads right (left) MARCH, Companies, HALT. " This places the men in Company fronts. The next command is " Cover Off. " Rear rank men cover off front rank men and blank files are eliminated. There are no file closers.

The Director then orders: " Front Rank, About FACE. " The two ranks are now facing each other. Three orders follow in rapid succession: " Ready, One Two. "

The men being at attention, the command " Ready " is to prepare them for what is to follow. At " One " the front rank man bends forward, grasping the legs of the rear rank man, just below the knees, at the same time placing his head between the legs of the rear rank man. The function of the rear rank man is simple. He merely wraps both arms about the waist of his partner, clasps the hands together and prepares to lift.

At " Two, " the men move in unison, the front rank man springing upward and the rear rank man assisting in the movement. The front rank man now throws his legs over the shoulder of the rear rank man to complete the position.

By this time the front rank man is upside down, his normal position having been completely reversed. The weight of the body is supported by three sources, namely (1) By the arms of the front man, who is now grasping the legs of the rear rank man; (2) by his own legs, which are hooked over the shoulders of the rear rank man; and (3) by the rear rank man himself, whose arms are wrapped about the waist of his partner. The head naturally is inclined to tilt-down, so the director commands: " Inhale, Exhale. "

The deep breathing is continued for two minutes, this time having been found to be most beneficial. When this period of time has elapsed, the commands are " Ready; DOWN. " The rear rank man bends forward and the uncoupling movement is executed in a manner just the reverse of the original. The front rank man, having regained his feet, the order is: " About; FACE. "

After a short rest the rear rank is turned about and the same procedure is carried out with this exception, the rear rank man is now the one who is lifted in the air.

The common fault is in mis-matched pairs, the front rank man, in some cases, being too heavy for the rear rank man to lift. In all instances it has been found that the men have been benefited immeasurably by the exercise. Free coughing has followed and the choked lung has been cleaned perceptibly.

### Ward Technic for Respiratory Tract Disease.

*At Hospital Center at Allerey.*

" 1. All gas, pneumonia and influenza and acute respiratory infections should be segregated and the wards should be cubicled.

2. The sheets should be at least 4 feet above and one foot below bed level, should extend at least 2-3 the bed length and be fastened to the wall.

3. Face masks should be made 8 by 5 inches, with at least 4 layers of gauze of good quality (24 $\times$ 20 strands or more to the square inch) with a tape one yard long sewed along upper and lower borders. The masks should be marked on the face side by a black thread tied on the gauze.

4. Masks should be worn by all physicians, nurses and attendants while on duty in the ward.

5. All patients should wear masks when out of cubicle. Fresh masks should be pinned on the cubicle sheet daily for all patients allowed to leave the cubicle. The medical personnel should hang masks on individual hooks in nurses' room.

6. Soiled masks should be collected daily, soaked in 2 per cent. cresol solution for one hour, boiled in soap suds, and dried. Each ward should care for its own masks.

7. Smoking is prohibited in these wards.

8. All patients should eat in cubicles, whether bed or ambulatory cases. Patients with masks on, however, may carry their own food or wait on others.

9. All dishes and eating utensils should be washed in boiling water.

10. All acute respiratory infection wards should be strictly quarantined, and marked " Contagious ". When a patient has passed the period of contagion (not less than one week of normal temperature) he may be removed to a Convalescent Ward.

11. In the Convalescent Ward cubicles should be used and masks for personnel, but not necessarily for patients. "

### Precautions Taken at Newport News.

(*Memorandum Order No. 8. Office of Surgeon, Port of Embarkation.*)

1. Most diseases are " hand-to-mouth " infections. The man with a mild unrecognized case of influenza or measles soils his hands with disease-laden saliva, and through his contact with things which he, as well as others handle, the disease germs spread to the hands of others; hence they are carried into the mouth, particularly at meal times.

2. It has been found that the number of cases of influenza in a command can be reduced about 80 per cent. by the use of boiling water for washing eating utensils. It is believed that this holds true for many other diseases, such as measles, scarlet fever, mumps and pneumonia.

3. It is, therefore, directed that all eating utensils be washed or rinsed in boiling water, and that all men wash their hands thoroughly with soap and water before each meal, and after visiting the toilet.

4. Wash water for tableware and mess-kits is in many instances not sufficiently hot to kill disease germs. Men with mild unrecognized cases of mouth-borne diseases, influenza, measles, pneumonia, etc., soil their hands and their mess equipment with germ laden saliva. These germs are washed off their hands and their mess equipment into the mess-kit wash water, and, if it is only tepid, the germs are not killed, but spread to the hands and eating utensils of other men. The mess-kit and the knife, fork and spoon must never be rinsed or washed in cold or tepid water. Swabs or mops will not be used as the handles will readily become contaminated.

5. The exact method of washing eating utensils and mess-kits is important. Three G. I. cans will be used. Two are permissible in an organization of 100 men or less. It is important that all these G. I. cans be placed over fire so that the water in them may be kept boiling. Each soldier will first scrape off his mess-kit and his knife, fork and spoon into a pail or other container. No water will be used in this process. He will then be required to plunge them into the first G. I. can, which will contain boiling water, preferably with soap dissolved therein; he must then rinse them in the second can containing boiling water but no soap, and then again rinse them in the third can. By plunging them two or three times in each can, one after the other, it will be found that the mess-kits are well cleaned. Mess-kits and eating utensils removed from boiling water will air-dry almost immediately. Drying them with a common towel is dangerous and will not be permitted.

If eating utensils — mess-kits or tableware — are washed by the kitchen force, they must first be placed in boiling soapy water, then rinsed off in boiling water.

6. Commanding Officers of Organizations will detail one officer to act as mess officer for each mess hall. Mess officers will be responsible for the cleanliness and sanitary conditions of the kitchens, mess halls, cooks and kitchen personnel, and will see to it that all kitchens and eating utensils, including mess-kits used in the preparation and serving of food, are kept clean, and washed as described above. The mess officer will also be instructed to take such measures as may be necessary to insure that all men wash their hands thoroughly with soap and water before meals. As the kitchen force may spread disease widely in an organization, any man of said force taken sick will be relieved forthwith and reported to the Surgeon. Particular attention should be paid to the cleanliness of the hands of all men detailed in the kitchen or in serving food.

7. This order applies not only during an epidemic, but at all times, and the success of this method of preventing disease, like all methods of precision, depends upon the thoroughness with which it is continuously carried out."

One is tempted to add that immaculate cleanliness of the hands and clothing of the kitchen personnel and an equal care in sterilizing the dishes in which food is served are of equal importance.

Short finger nails and scrubbed hands in the kitchen mean clean food on the table.

### INFLUENZA.

The pandemic of influenza in Italy, Switzerland, Hungary, Germany, Portugal, South Africa and Cape Verde Islands.

Rome, Italy, October 21, 1918. We give here a summary report of Comm. Alberto Lutrario, General Director of Public Health, presented at the recent meeting of the High Council of Health.

"The infectious disease which has invaded a large part of Italy is only a form of the pandemic which, after raging in America, has now for some time been traveling throughout Europe.

"It penetrated into our country, where some very serious cases of contagion have occurred, because of the disease's natural and extraordinary tendency to spread, and because of the present special circumstances which have turned Italy into a public thoroughfare.

"Influenza is a very old disease, mention of which is found to have been made as far back as the fifth century B. C. As many as 128 epidemics of influenza have been carefully studied to date, nor can its appearance in summer give place to doubt as to its identity, for it is known that 35 epidemics have taken place in spring, 24 in autumn and 17 in summer.

"The disease showed itself among us as early as last spring, first by sporadic cases which passed almost unobserved, and later by small epidemics which broke out at Assisi, Domodossola, Spezia, and the Provinces of Modena, Piacenza, Verona, and Pisa.

"In June it spread to the provinces of Baria and Taranto: in the first part of July, when this first phase may be considered to have passed, it broke out in a more violent and serious form in Calabria. Starting from Rosarno, it spread rapidly to the province of Reggio, Alessandria, Torino and Liguria.

"In September it spread throughout all the provinces of the Kingdom with varying amount of contagion and proportion of deaths.

"The provinces which suffered most were those of Palermo, Catania, Caltanissetta, Foggia, and Bari, where the disease, however, is now clearly on the wane.

"In Latium, Abruzzo, Piedmont, and Lombardy, which were attacked later, the epidemic may be said to be approaching its highest point. Finally, in Veneto and Liguria it still persists in the form of sporadic cases.

"The rate of mortality of the disease has been greatly exaggerated, for, even in the localities worst hit, it has reached only one, two, and very seldom, three per cent. of the cases. The contagiousness, on the other hand, has been very high.

"Concerning the manifestations of the disease, the clinical record shows that it starts suddenly in the form of chills, fever, and pain in the throat and nose, accompanied by smarting; then headache follows, pain in the loins, annoying cough, and an intense general weakness. This condition (subject to alternations during which the nose sometimes bleeds and the fever rises as high as 40 degrees C.) lasts three or four days, after which the patient becomes convalescent but continues to feel indisposed for several days.

"There are light cases in which the fever disappears after the second or third day, and serious cases in which broncho-pulmonary complications follow and death may result.

"The time the disease lasts varies with the region."

Budapest. "Neue Freie Presse" (for Oct. 16.) — "Yesterday 1,400 new cases of Spanish Fever were reported to the Central Office. In this number there are not included the numerous cases received by the various hospitals; with these the total number of the cases amounts to 1,574. Yesterday 62 persons died as a result of this sickness."

Berne. "Zuercher Zeitung" (for Oct. 17) — "Since the number of cases of pulmonary plague is again on the increase the Sanitary

Commission has renewed the order against the holding of any meetings whatever."

Basel, October 16. "The Health Office of the Canton reports that the number of cases in the week of October 6th to 12th was 3,237, as compared to 1,613 in the week before. In this number are included 1,331 babies (737 in the week before) and 1,906 adults."

Geneva. "Zuercher Zeitung" (for Oct. 16) — "The Council of the Canton has issued an order prohibiting all persons under 18 years of age from attending moving picture performances, theatres and public meetings, even if accompanied by relatives. No performance can last more than 2 hours."

Wiesbaden, October 14th. "Because of sudden increase in the cases of illness all schools were closed for 14 days. The Postal Department was compelled to limit the mail service because of a lack of personnel."

The Hague. "Frankfurter Zeitung" (for Oct. 15th.) — "The cases of sickness are again on the increase. Yesterday the number of deaths was 250. The number of deaths from October 1st to 10th 1500."

Swinemuende. "Koelnische Zeitung" (for Oct. 15th.) — "It is reported from numerous cities of the provinces of Pomerania, Stettin, Greifswald, etc., that the Spanish sickness is playing havoc. In many cases the postal and railway service stopped because of the lack of personnel. The percentage of deaths is comparatively large."

Lisbon, October 23. "On the 20th of October, 1918, the steamship "Mozambique" arrived in Lisbon from Mozambique and Cape Town. During the voyage, an epidemic broke out on board, causing the death of 200 of the 800 passengers. Within 24 hours of the ship's arrival in Lisbon, 26 more deaths took place.

"Among the passengers there were 558 soldiers returning from South Africa. 177 of these died from the pneumonic fever, it is said.

"The deaths increased after the steamer left Cape Town, where the epidemic was raging and where hundreds were its victims.

"Here in Lisbon the situation appears to be unchanged, though it is reported that the number of cases entering the hospitals has diminished somewhat.

"The influenza, called in Portugal the pneumonic influenza, is of a particularly malignant nature, many of its victims dying within 48 hours afer the attack.

Lisbon, October 31st. "In Setubal the epidemic is causing many deaths.

" A statement by the Director General of Sanitation reveals the fact that 25,000 people have already died of this disease in Portugal, and that an average of 300 are dying daily in Lisbon.

" The epidemic has attacked the greater part of the population of the Cape Verde Island, and has greatly increased the miserable condition of the natives. "

Up to the middle of October there had been 200,000 cases of influenza among our troops in the United States with a very high incidence of complicating pneumonia and a high case mortality rate. It is estimated that almost as many more cases and deaths have occurred since then.

The height of the epidemic in the American E. F. was reached in the week ending October 27th. Since June 15th (up to November 8th) there have been 78,713 cases of influenza, and 11,165 cases of pneumonia, almost all complicating cases of influenza. There have been 191 deaths attributed directly to inflenza, and 4,754 deaths from pneumonia in this period. At the height of the epidemic there were 10,983 cases of influenza in one week ending October 25th, and the case mortality from pneumonia (1,960 cases) rose to 75 per cent., in the last week for which completed records are available (week ending November 8th) there were 5,972 cases of influenza, and the case mortality for pneumonia (1,062 cases) fell to 18.5 per cent.

In spite of the generally prevalent opinion that this epidemic has been due to Pfeiffer's bacillus influenza, there is so much accurate and well considered bacteriological and pathological work which casts doubt upon this assumption that it behooves us to be conservative in our opinion as to specific etiology of clinical influenza, and to continue well controlled clinical, bacteriological, pathological and epidemiological studies on the matter.

Bulletin No. 5, Hdqrs. Base Section No. 2, S. O. S., A. E. F., issued from the office of the Base Surgeon November 11th, which can doubtless be obtained on request, gives an admirable description of the epidemic as seen and studied in the area about Bordeaux.

In the Medical Supplement for October 1, 1918, issued by the Medical Research Committee, there is given on pages 354-360 a comprehensive summary of recent clinical and bacteriological information which indicates the complexity of the still unsolved problems of etiology and epidemiology of this disease.

### ACUTE RESPIRATORY INFECTIONS.

*Extracted from Bulletins on Transmissible Diseases Published by the American Red Cross in France for the Division of Laboratories and Infectious Diseases of the American Expeditionary Forces.*

### General Regulations for the Care of Infectious Diseases in Hospitals.

As far as possible separate wards for the different infectious diseases should be established. When this is impossible for lack of space, beds should be screened from those adjoining, either by improvised frames or by wires stretched across wards over which sheets are hung. These regulations apply to pneumonia, meningitis, measles, scarlet fever, diphtheria, small pox, chicken pox, and mumps. Pneumonia of any description is to be dealt with as a transmissible disease and included in all such precautions.

The strictest attention should be paid to ventilation of the wards containing patients, both day and night, but draughts should be avoided and the wards kept reasonably warm. Floors should be oiled when possible and mopped once or twice a day to prevent dust. Dry sweeping should be rigidly prohibited.

At least 5 feet should be allowed between beds; but where crowding cannot be avoided, patients should be so arranged that one man's head is placed opposite his neighbor's feet.

Separate thermometers, bed pans, eating utensils, and other material should be used for each patient wherever possible, and careful sterilization after use rigidly enforced. Dishes should invariably be boiled.

Bed and other linen should be soaked in a disinfectant solution before being sent to the common laundry.

Nasal and oral discharges should be burned or disinfected. In the case of typhoid fever and other intestinal diseases, feces should be incinerated and bed pans soaked in disinfectant solutions.

Measles should never be permitted to remain in the same wards with pneumonia or severe respiratory infections of any kind. Measles pneumonia should be removed from measles wards as soon as pulmonary involvement is recognized, Measles pneumonia and primary pneumonia should never be left together. In both measles and pneumonia of any description beds should invariably be screened one from the other so that cross infection may be avoided.

*Attendants.* — Attendants and nurses assigned to the care of infectious diseases should be chosen as far as possible from those immune to the respective diseases. Whenever possible separate attendants should be employed for the different diseases and such attendants should be separated from other personnel as far as sleeping quarters and mess are concerned.

Schick tests should be done on attendants taking care of dipheria cases, and only those showing negative reactions should be employed. Periodical cultures should be taken from attendants caring for meningitis and diphtheria cases, and two negative cultures taken not less than 24 hours apart should be obtained before this personnel is released for the care of other patients.

Under no circumstances should the same nurse or attendant take care of measles and measles-pneumonia at the same time.

The careful washing of hands should be enforced before doctors or nurses leave the wards.

Gowns will be worn in the wards. Masks may be used in meningitis wards and wards containing respiratory diseases, scarlatina, measles, and mumps.

Attention is called to the fact that serious sequelae are likely to develop during the convalescent periods of infectious diseases, and therefore the return to normal routine should be carefully graduated with especial attention to feeding and warmth at night.

No cases should be discharged until two negative cultures have been obtained — in diseases in which such examinations are possible.

Special examinations of the urine should be made before discharge, and no case of infectious disease should be released until the urine is albumin-free.

In the case of measles and scarlatina, no case should be released until all discharges have stopped and no signs of catarrhal inflammation remain.

### Measles.

As regards mode of conveyance and danger to life, measles is to be looked upon as a respiratory disease. It is conveyed by the secretions of the mouth and nose. The dangers of infection are therefore associated with coughing, sneezing, and spitting, and the indirect conveyance of saliva and mucus with handkerchiefs, etc. These secretions are infectious during the pre-eruptive stage, and since individuals developing measles usually have catarrh of the upper respiratory passages, they will have thoroughly exposed their surroundings by the time the eruption has appeared. The incubation period is usually ten to fourteen days.

In consequence, every individual living in the same barracks or billet, or in any way closely associated with the patient during the week preceding the diagnosis of measles, must be regarded as a possible contact.

It is always advisable to take the following precautions, since these will undoubtedly diminish incidence.

1. The patient should be immediately isolated. His clothing and blankets should be removed and disinfected.

2. The entire company or barrack population should be inspected for rash, injection of the conjunctivae, and colds in the head. This should be done daily for ten days. Temperatures should be taken on all suspicious cases.

3. Contacts and men having conjunctivitis and colds in the head should be segregated as to mess and quarters, and drilled and worked in separate units.

4. Tent-mates or men sleeping next to and above patient should be segregated as to sleeping quarters and mess arrangements and carefully inspected every day, this to be continued for two weeks. In all other respects they should be allowed to do duty unless they are coughing and sneezing.

5. Promiscuous spitting should be made an offense subject to disciplinary measures.

6. Attention should be directed to thorough ventilation of quarters, day and night, and bedding and blankets of men should be spread in the sun whenever possible.

*Measles in Hospital.* — The danger of measles to the patient is chiefly that of pulmonary complications. For this reason, measles cases should be kept in separate wards with five feet between beds. Screens made of streched wires or frames over which moist sheets are hung should be arranged between beds (sheets of newspaper on frames can be used for this). Every effort should be made to protect the patient from droplet infection by the coughing of other patients. Floors of ward should be mopped frequently with creolin or liq. cresol. comp. or other disinfectant solution, or oiled to prevent dust. The greatest attention should be paid to the care of mouths of patients. Sputum and nasal discharges must be disinfected or burned. Eating utensils should be boiled. Individual drinking cups should be used and disinfected. Nurses and doctors should wear gowns while in wards and should wash faces and hands on leaving it.

Measles cases that develop pneumonia should be immediately removed from the measles ward. They should not be placed in the same ward with primary pneumonia. If possible they should be isolated or put into a separate measles-pneumonia ward.

Convalescence is one of the most important periods of measles. Many pneumonias are acquired during this stage. It is essential that at least two weeks from the time of fading of the rash should be allowed for convalescence. During this period especial attention should be paid to keeping the convalescent warm and protecting him from exposure, while giving him as much fresh air as possible. Return to activity should be gradual, and complete physical and urinary examination should precede discharge from hospital.

### Lobar Pneumonia.

Pneumonia in military organizations must be regarded as an infectious disease which is conveyed from individual to individual with saliva, sputum and nasal discharges. The only reason that pneumonia does not more often occur in widespread epidemics is the fact that the healthy and well-nourished human being possesses a high normal resistance to pneumococcus infection. In the prophylaxis of pneumonia the most important factors are the prevention of, first, those circumstances which lower this resistance, and second, those which facilitate the conveyance of secretions of mouth and nose from one person to another. The most dangerous source of spread of pneumonia is the patient who is throwing out virulent pneumococci with his sputum. However, a considerable number of normal individuals carry virulent pneumococci in their mouths and throats.

Ordinarily the pneumococci carried by normal individuals are of relatively low virulence — the so-called group IV organisms. But even these will cause pneumonia in individuals who are in a state of low resistance. However, there are also normal carriers of the more virulent organisms of groups I, II, and III, these being often individuals who have been in contact with patients having such infections. In military epidemics so far studied, all groups of pneumococci have been represented, and it is likely that the susceptibility factor is the one of the greatest importance.

Epidemics of pneumonia in armies are most likely to occur among unseasoned troops during the cold weather. When large numbers of men develop " colds " and bronchitis, the general susceptibility is increased, the distribution of sputum becomes more general, and the number of carriers of virulent pneumococci is increased. Thus a vicious circle is established. The most important sanitary prophylactic measures to be attended to when cases begin to occur are :

1. Overcrowding in barracks, tents, or billets must be corrected.

Distance between beds, i. e., floor space, is more important than actual cubic capacity.

2. Ventilation of sleeping quarters must be attended to.

3. The men must be protected from unnecessary exposure. Mess halls must be warm. Clothing and blankets should be sufficient. Arrangements must be made for drying of clothes, socks and shoes when returning from duty. Overwork of young troops must be avoided.

4. Careful attention must be paid to men with colds and coughs at sick call and temperatures taken on the more severe ones. Men who are coughing severely and sneezing should be temporarily segregated in sleeping quarters.

5. Promiscuous spitting should be suppressed.

The most important single factor which leads to the spread of pneumonia is overcrowding in barracks.

In hospitals pneumonia patients should be treated as infectious for others and segregated in special wards. Primary pneumonia should not be treated in the same wards. Strict attention should be paid to oral hygiene, and thermometers, eating utensils, etc., should be sterilized. Cases in wards should be screened from adjoining beds to prevent cross infection.

Where facilities exist type determination should be made on the sputum of all cases as soon as possible. Where such facilities do not exist the nearest well equipped laboratory should be appealed to.

If a type I organism is found, a skin test for hypersensitiveness to horse serum should be done before serum is administered. *Serum should never be administered unless type determination can be made.* It is only in Type I cases that serum has been found useful, and to give serum in other type infections not only wastes the serum but uselessly renders the patient hypersensitive to future injections of horse serum.

Pneumococcus serum is given intravenously in doses of 50 to 100 cc. It is best given diluted with an equal volume of salt solution in order that slowness of administration may be facilitated. The entire process should consume at least 20 minutes, the first 10 cc. being given as slowly as possible (1 cc. per minute). The slightest signs of anaphylaxis should indicate immediate interruption of the injection. (See circular on Serum Administration and on Anaphylaxis, p. 8.)

*Non-Specific Treatment.* — *a.* It is advisable at the outset to put the heart well under the influence of digitalis, irrespective of the quality of the pulse. Digitalis is slow in its action, and if the administration is begun only after the need is apparent, it may be too late.

The best form of digitalis is the Nativelle's pill of 1/4 milligram (grain 1/250). This is the French preparation of crystallized digitaline. Four of the pills may be given by mouth in the first 24 hours, and four in the second. When these pills are not obtainable digipuratum may be employed at the rate of one tablet every five hours until ten are given.

Should neither of these forms of the drug be available the tincture may be used at the rate of twenty-two minims for each tablet of digipuratum.

If, after two days' interval without the drug, the pulse becomes very rapid, digitalis may be repeated cautiously at the same rate of administration with a view to reducing the rate of the pulse.

*b.* For severe coughing which exhausts the patient, and for severe pains of pleuritis, morphia is necessary. Intractable abdominal distention is sometimes relieved by pituitrin. Strychnia, so commonly used as a stimulant, is without demonstrated value in pneumonia and should not be given. When collapse occurs, camphorated oil may be used.

Fresh air is an important factor in the treatment of pneumonia. The patient should be kept warm, but the windows should be open and the room thoroughly ventilated, irrespective of the outside temperature.

### Meningitis.

The seriousness of epidemic meningitis as a military disease, and its likelihood of spreading in men of the age-group composing our army, render it desirable to establish uniformity of procedure in regard to sanitary and therapeutic measures, as far as this is consistent with military conditions.

*Sanitary Principles.* — The meningococcus is probably conveyed with the naso-pharyngeal secretions of a case or a carrier, and gains entrance to the body of the new host by way of the upper respiratory passages. Since the micro-organism does not survive for any great length of time in the outer air, the danger of infection is chiefly direct — from person to person. Perfectly healthy individuals may carry the virulent organisms for months and even years.

Since the percentage of such carriers in military communities has been found to vary anywhere from two to ten or more percent., according to season and the prevalence of the disease, it is obvious that much opportunity for the acquisition of the meningococcus exists. It is also plain that this danger is greatly increased when colds and bronchitis increase the amount of spitting, sneezing, and coughing.

The conditions which favor spread of the disease, therefore, and against which sanitary attack should be directed are :

1. Overcrowding in sleeping quarters.
2. Faulty ventilation of sleeping quarters.
3. Promiscuous spitting and coughing.

In the prophylaxis of this disease, as well as of the various respiratory diseases, the most important preventive measures are those which aim at the protection of the men from " catching cold." A common cold increases the distribution of saliva of carriers and increases the susceptibility of the recipients. While " colds " cannot of course be wholly prevented, medical and other officers should remember that much may be accomplished by attempting to enable the men to have dry socks and clothes when at rest, dry beds, and warm clothing and blankets. A man with a severe cough isolated from others for a short period may mean the prevention of a dozen similarly disabled. It is emphasized that the most important sanitary measures in treating this disease consist in increasing the floor space between beds, the ventilation of quarters day and night, the airing and sunning of bedding, and the cleanliness of floors. When the carrier rate is above 10 o/o, or when several cases have occurred in a command, especial care in these matters should be exercised, and at least 5 feet should be allowed between bunks.

When a case of suspected meningitis is discovered, the following steps should immediately be taken :

*The Patient.* — 1. The patient should be immediately taken to a hospital and properly reported. The case should be isolated, if possible. If not, the bed should be screened against those adjoining. When several cases occur, a separate meningitis ward should be established whenever possible.

2. Lumbar puncture should be at once performed and the fluid submitted for diagnosis to the nearest laboratory.

3. Intraspinous injection of polyvalent antimeningococcus serum should be carried out without delay, not waiting upon laboratory diagnosis unless this can be obtained on the premises and at once.

When the puncture fluid is perfectly clear, careful re-examination of the case is justified before serum is administered, but it must be remembered that recently cases have been observed in which first puncture has yielded clear fluid, later specimens being typically purulent. The first intraspinous dose of serum should usually consist of about 30 cc. Since there is reason to believe that meningitis is often, in its origin, a bacteriemia, it is a good plan, at the same time, to give 40 to 60 cc. intravenously, administered with great slowness and with vigilance for signs of anaphylaxis (i. e. respiratory distress, collapse).

When a positive bacteriological diagnosis has been received, at least two additional doses of 30 to 50 c c. each should be given intraspinously on succeeding days unless clinical reasons contraindicate.

Routine blood cultures should be done on all suspected and diagnosed cases. Male attendants should, if possible, be segregated from other personnel as far as mess and sleeping quarters are concerned. Nasal and oral discharges of the patient should be disinfected — best burned. Care should be exercised in the separate sterilization and washing of eating utensils. Nurses and doctors should exercise great care in the washing of hands on leaving cases and should wear gowns while attending them. Whenever feasible, periodical carrier examination should be done on all hospital personnel in contact with cases. Convalescent patients should be kept isolated until 3 carrier examinations have been negative at intervals of from 3 to 6 days.

*Contacts.* — When a single case of meningitis is discovered in a command, contacts should promptly be segregated. It does not seem possible to require carrier examinations for sporadic cases in the A. E. F. as a routine measure.

The definition of a contact must vary according to prevailing conditions.

If the command is living in tents, all tent-mates are considered as contacts. If the command has been living in billets, all men sleeping in the same room are considered as contacts and a painstaking effort should be made to discover any other comrades who have slept in the same room with the patient within two weeks. These should be added to the contact group. If the command has been living in barracks, those sleeping on either side and above the patient at any time during the last two weeks should be considered contacts, as well as chums or intimate friends who are known to have spent much leisure time with the patient.

If two or more men of the same mess, same barrack, tent, or billet develop meningitis, carrier examinations should be done on a "sample detachment" of 25 men, chosen at random from different platoons, to determine the "carrier rate" for the company. Another detachment of 25 men similarly taken from another company of the same regiment should at the same time be swabbed to determine the "non-contact rate" of the regiment.

If two or more cases occur in the same barrack, similar " rate " determination should be done with this barrack instead of the company as a unit.

Companies or barrack population in which two or more cases of meningitis have occurred should be segregated from the rest of their commands as far as eating and sleeping are concerned, but otherwise allowed to perform duty.

When the " carrier rate " of any command is found to exceed 10 o/o, special measures aimed at the reduction of this rate, such as steam spray rooms, individual spray treatment, etc., will be instituted under the direction of the Chiefs of the Sanitary and Laboratory Divisions, details of which will be worked out in each instance, according to the possibilities for such work determined by the participation of the unit in military operations.

*Carriers.* — Carriers will be segregated from other troops as regards mess and sleeping quarters. They may drill and perform other outdoor duties as usual. These may, if necessary, be performed together with other troops in company formation, though it is desirable whenever possible to keep them together as a separate detachment. Especial care will be given their quarters to avoid crowding and ensure ventilation, and their mess-kits will be boiled after every meal. Carriers with severe coughs and colds will be specially segregated and excluded from contact with others. Promiscuous spitting by carriers will be made an offense subject to disciplinary measures.

Treatment of carriers by steam inhalation rooms and sprays will be instituted under direction of the Chief Division of Sanitation, and swabs will be taken at intervals of from 3 to 6 days. Temporary carriers will be returned to regular duty with their commands when 3 negative examinations have been reported. Individuals who remain carriers for one month or longer will be regarded as chronic carriers, and made the subject of special measures to be determined on the basis of their numbers and other attendant circumstances by the Chief of the Division of Sanitation.

*Laboratory Diagnosis.* — Spinal fluid submitted for diagnosis should immediately be divided into two parts. One part should be placed in the incubator at once, the other centrifuged. On the sediment of this portion a Gram stain should be made. Gram-negative diplococci, intra or extracellular, of course determine the diagnosis. It should be remembered, however, that in many fluids meningococcus are few and difficult to find and undergo rapid autolysis. In such cases fluids with considerable numbers of polymorphonuclear leucocytes should be considered as tentatively positive, in the interest of speed in treatment. In other forms of acute meningitis, such as pneumococcus and streptococcus, the bacteria are usually present in relative abundance, and in a purulent spinal fluid in which no bacteria can be found in smear, the eventual diagnosis is usually meningococcus.

The incubated portion of the fluid should be planted in quantities of from one to two cubic centimeters on plates of laked blood glucose-agar. In all cases isolations of the cocci should be done in order that subsequent type determination can be made. It is always well to preserve a part of the original fluid in the incubator for 24 hours, since the cocci may increase in such fluid when growth on the plates does not develop. It should be remembered in all cultural work with meningococcus that this organism dies out rapidly at room temperature. Delay in the incubation of spinal fluid and of cultures should therefore be avoided.

*Therapeutic Measures Other than Serum Treatment.* — *a.* Drainage of the meninges is important. At each puncture allow the spinal fluid to escape so long as it flows freely.

*b.* In cases of extreme restlessness and loss of sleep morphia may be given, except when associated with increased tension and marked lowering of the respiration; then use chloral hydrate, in doses not to exceed 0.65 gram (grains X).

*c.* Be careful to guard against the inhalation of food, especially of fluids. Turn all dull or delirious patients on the side before feeding, and give the food slowly in small amounts. Be sure that each mouthful is swallowed before the next is given.

*d.* The mouth should be washed with a suitable antiseptic solution (Dobell's or Listerine or the like) before and after each feeding. It is well to grease the lips daily.

*e.* Be as careful to avoid bed sores as in typhoid fever.

